# Progress in Medicine

**Published:** 1896-05-16

**Authors:** 


### Progress in Medicine.

### INFECTIVE DISEASES.

(*Continued from page* 59.)

**Tropical Fevers.**—The rapid growth of the British Empire in Southern and Eastern Africa, in Ashantee, and among the fifteen millions of the Housa peoples, entails an immense and increasing mortality from these diseases among men of English race. Unfortunately, the affections produced by plasmodia, such as malarial fevers, as opposed to those resulting from bacteria, are not affected by modern advances in treatment by serum or antitoxins, though there are other methods available. Our knowledge of these plasmodial diseases is very backward, but now advancing. Manson's theory that the germ of malaria finds an alternate host in the mosquito, gets much support from the known transportation of the filaria which has been proved to be carried in a similar way by mosquitos.[1] So, too, the plasmode of Texas fever in cattle appears to be carried by or to infest the cattle ticks ; and recently D. Bruce has shown that the terrible "tsetse fly" disease is caused by an infusorian which is carried in the fly from one animal to another. It is probable that this last parasite is the same as those of the Indian "surra" disease in horses. They are actively motile, and destroy the blood corpuscles in enormous numbers, while they may themselves amount to 300,000 in the cubic mm. Though inoculation seems useless, arsenic has had some effect in the few cases in which it has been tried. In the treatment of malarial fevers quinine still remains the most important remedy and prophylactic we have. J. Harris[2] writes, however, against its abuse, viz., the carelessness of giving it in fevers which are not really malarial, of giving it in excessive quantities, or to very anæmic patients. Other writers attribute great destruction of red corpuscles and various disorders to its inordinate use. However, the malarial parasite in its early stages is quickly killed by a little quinine in the blood, though it becomes more resistant as it grows older.[3] The chief need is for some method of administering quinine in sufficient doses without irritating the stomach, or without producing abscesses if given subcutaneously. G. Thin[4] recommends daily doses of five grains as a prophylactic, and gives numbers of instances where men on the West Coast of Africa remained free from fever when taking this dose regularly from two days before entering the district until ten days after leaving it. Maurage[5] has employed with success in a few cases the drug Calaya obtained from the Annεslea febrifuga, which he thinks produces no sweating or depression. Among the more severe forms of the disease Manson[6] notes the occasional fatal hyperpyrexial attacks which are often mistaken for sunstroke. There are also algid choleraic ones, which for the time exactly resemble severe cholera. Another most severe type found only in a few large districts but increasing in its area, and which leaves those who survive so weak and with kidneys so damaged that they often die under any new complaint, is the blackwater fever. It generally attacks a patient about the third year of his residence in the malarial district. Immense quantities of pigment and tube casts are passed in the urine, but few or no red corpuscles. Much pain in the loins and liver, with vomiting and jaundice, are complained of, and the question arises whether it is due to a special organism. Manson leaves this undecided, but an American, Wolderl,[7] gives an elaborate account of the form which he met with in a case of malarial hæmaturia with some beautiful plates, showing the crescent and other well-known types.

An interesting discussion took place at the Royal Medical and Chirurgical Society on the varieties of the parasite. Marshall and Thin[8] took the position that there are probably as many varieties of parasites as there are kinds of fever, and they pointed out minute differences in pigmentation and in the changes in the cells attacked, which they thought were produced by different organisms, and marked by different clinical features. Professor Cayley[9] took the extreme view on the other side that every form is but a modification of the same parasite, and that there is only one species of malarial fever.

With the exception of Surgeon-Major Lawrie, there is almost universal agreement as to the importance of the plasmode. Maynard,[10] indeed, in India could only find it, after a careful search, in a quarter of his cases of malaria. This is very different to the constant occurrence of the parasites in every case, which American observers report, but that this is not due to local peculiarities is clear, since Ross,[11] at Secunderabad, found them much more frequently even in a hurried examination, and noted that the Italian classification held good for India. It is quite certain that the plasmode has other hosts besides man, for it is common in forests when man penetrates for the first time, and Manson[12] shows strong reason for belief that the flagellated forms are not degenerate ones, but special adaptations to carry on its life outside the human body. Thus Ross watched the adventures of a flagellum for three hours as it swam about, and fought with its neighbours, and when malarial blood was swallowed by a mosquito, he saw the crescents in it quickly become spheres, and develop flagella about twenty-five minutes afterwards. These and many other observations lend much probability, though they do not as yet prove Manson's theory that the malarial plasmode finds its more usual host in the mosquito.

Charles Finlay,[13] on the subject of yellow fever, brings forward much evidence to show that the M. tetragenus is the active agent in yellow fever, that it is in some cases transported by the mosquito, and that protection can be obtained by certain mosquito inoculations. Sternberg,[14] however, insists that the bacterium is a common saprophytic one, and has nothing to do with yellow fever.

**Small-pox.**—There appear to be rare cases of hæmorrhagic variola which do recover, though the great majority are fatal. Whether these recoveries are due to the effect of early vaccination is not clear. One such instance in a man of twenty-one is recorded by Ricketts and Frith.[15] After profuse hæmaturia and other forms of bleeding all his bad symptoms disappeared. Another case in a pregnant woman is given by F. Robinson,[16] where a severe attack was similarly arrested, and not even the pregnancy was interrupted. Annet[17] records an instance of intrauterine transmission of vaccine immunity. The patient's mother underwent small-pox while she was pregnant, and repeated vaccinations of the patient failed during his childhood. The immunity disappeared in later life, and he was then successfully vaccinated. Annet notices[18] that the immunity thus obtained through maternal influences is usually of short duration, and records an instance where vaccination itself during pregnancy produced immunity in the infant. A curious case of inoculated or local variola occurred lately in Germany. Wagenmann[19] had a partly-protected patient, who was nursing a sister sick of variola, and splashed some of the bath water into her own eye, whereon pustules appeared locally, and a mild attack of variola followed. The outbreak at Gloucester has attained such proportions as to occasion great loss of life as well as terrible distress in all classes. Up to April 23rd 1,470 persons had been attacked. It was noted that of the first 365 cases at the hospital 207 were in non-vaccinated persons, and all but one of the rest were in persons once vaccinated in infancy, while of the 81 deaths 70 were in non-vaccinated persons and four in those once vaccinated. These results have followed after the Vaccination Acts have been allowed to lapse for nine years only.

**Scarlet Fever.**—Marmorek[20] has reported the treatment of 96 children suffering from scarlet fever by antistreptococcal serum. The lymphatic glands quickly decreased in size, the exudation in the throat was cast off, and the pulse became full and strong. In puerperal fever this[21] same serum was also used in five severe cases with brilliant results. The good effects, however, were much more marked in respect to the general septicæmic phenomena than in the case of abscesses and other local manifestations. Similarly in erysipelas Chantemesse found the death-rate in 500 patients treated with serum of various strengths to be $2\frac{1}{2}$ per cent., while with symptomatic treatment it was only $3\frac{1}{2}$.

Rommelaere[22] has also reported successful results with some powerful serum made at Louvain. Two cases of peritonitis after operations were cured, and generally in coccal diseases rapid amelioration of the symptoms took place after injection.

**Cholera.**—So lately as January last a ship came to anchor at Holyhead[23] with cholera on board, which was probably contracted at Constantinople. Two deaths occurred, but the disease then ceased to spread. In Egypt, on the other hand, some nine hundred and fifty deaths have taken place from cholera during the winter,[24] and much anxiety will be felt as to the possibility of stamping it out before the hot weather comes. One result of the Indian cholera investigations has been the appointment of a committee to revise the measures ordered when cholera breaks out among British troops. Mr. Hart suggests that sterilisation of drinking water and disinfection of all excreta are the chief means of stopping the spread of an epidemic.[25] His views as to the spread of cholera by contaminated water are supported by Dr. Banks, the late medical officer of Puri, in Orissa, the centre of much of the pilgrim traffic. The latter[26] points out that the evidence is strongly against atmospheric communication, but that the disease spreads along the lines of human traffic generally through infection of the drinking water, and, moreover, that the period of incubation after infection is nearly always under three days. These views he supports by analyses of the outbreaks which occurred at some of the recent pilgrim fairs. C. A. Gordon,[27] another Indian officer, remarks, however, that of different groups of persons using the same water supply some will be attacked and others escape; and, again, certain villages along a line of road will escape while the rest are infected; and, in short, that no theory so far seems to meet all the facts. As to the toxin of cholera Ouchinsky[28] shows that the bacteria can produce it in media free from proteids, and that it is, therefore, not a result of decomposition of proteid matter. Chemically it appears to be allied to the nucleo albumens.

**Hydrophobia.**—The argument in favour of the Pasteur treatment is given briefly by Shirley Murphy[29] as follows. Before it began out of every hundred bitten from 20 to 70 died. Now 17,000 have been treated at the institute, and the total deaths, according to the anti-vivisectionists, have been 283; but on the expectant treatment there would have been at least 20 per cent., or 3,400 instead of 283. But they assert that the treatment causes hydrophobia. If so the deaths should be 3,400 (the natural rate), plus the cases caused by the treatment, and yet on their own showing these are only 283, or 1·66 per cent. Pasteur, indeed, reckons the deaths as 81 only. On the other hand, Benham[30] denies the value of the evidence that 20 per cent. of those bitten by mad dogs perish, and points out that this rate would mean 300 deaths a year in France alone before 1880. Babes[31] has written an elaborate paper on the pathology of the disease, in which he lays stress on the presence of certain hyaline bodies in the nerve cells, glands, and vessel walls, which are a valuable test for the disease.

**Anthrax and Actinomycosis.**—Marchoux[32] has investigated the relative value of serum and vaccination by small quantities of the virus in the treatment of anthrax, and finds the effect of the latter more complete and lasting. Kanthack[33] has unearthed some drawings of specimens of actinomycosis made by Mr. Thomas Smith, of St. Bartholomew's, in 1855, or twenty-three years before Israel brought the disease before the notice of the world. Smith, like Langenbeck, who made similar sketches in 1845, did not understand or publish his discovery, though he wrote a clear account of the tissues and the "cells looking a little like starch granules, but having radiating lines running from their circumferences." Hummel[34] finds that there are only twelve cases reported in which the mode of infection was known. This was in every instance a foreign body. Grill gives an analysis of 107 cases of actinomycosis affecting the digestive tract, which appears to be the most frequent seat of the disease next to the head and neck. He agrees with Hummel that cattle and men probably catch the disease from some source common to them both, such as raw grain which happens to wound the mucous membrane. Diarrhœa, pain, and other symptoms may be followed by an abdominal tumour which discharges externally, leaving troublesome fistulæ behind it. Recovery took place in a third of the cases.

**Leprosy.**—Carrasquilla[35] claims that he has found a serum which cures not only nervous, but tubercular leprosy. Anæsthesia disappears, œdema and ulceration pass away as well as the tubercles, while the general health rapidly improves. In his fifteen cases the author found these changes followed the use of the serum without fail; nor was there so far any fresh development of the disease in them. Much interest will be felt in the forthcoming paper on the method of preparation of this serum. In connection with this matter we may mention Chantemesse's[36] method of administering serum in various febrile diseases per rectum. He finds that the results are as good as when given hypodermically, and, so far as he can judge at present, the dose required is equal under either method. He employed the plan with success in twenty cases, including diphtheria, erysipelas, &c. D'Arsonval and Charrin announce also the modification of toxins by electricity[37] in place of filtration or heating. Thus antitoxins were prepared, and animals to whom lethal doses of diphtheritic or other poisons had been administered were preserved when certain forms of electricity had been passed through their bodies.

The receptivity for various diseases, or the likelihood of being attacked when exposed to infection, makes a great difference in the manner in which an epidemic spreads. Goffstein[38] calculates that of 100 children exposed to measles 95 will take it. So, too, of 100 exposed to scarlet fever the expectation equals 40, while for diphtheria the factor is only 10. The curve of the epidemic varies in a similar way; thus, that of measles is very short, while that of diphtheria lasts at least 25 years, and the period of its return is proportionately deferred.

[1] Nature, April 16. [2] N. Y. Med. Jour., Feb. 1. [3] Lanc, Feb. 1. [4] Lanc., Jan. 25. [5] Lanc., Jan. 11. [6] B. M. J., Feb. 1. [7] Jan. 4. [8] Lanc., Feb. 15. [9] Lanc, Feb. 29. [10] Med. Rec., Jan. 4. [11] B. M. J., Feb. 1. [12] B. M. J., March 14. [13] Edin. Med. J., Dec. [14] Edin. Med. J., March. [15] Lanc., Feb, 1. [16] Lanc., Jan. 4. [17] B. M. J., Feb. 8. [18] Med. Rec., Jan. 4. [19] Int. Med. Mag., Dec. [20] Lanc., Feb. 22. [21] Practitioner, March. [22] Med. Week, Jan. 3. [23] Lanc., Jan. 25. [24] Lanc., Feb. 22. [25] B. M. J., Jan. 4. [26] Glasgow Med. J., March. [27] Lanc., Feb. 1. [28] Amer. M. S. Bull., Nov. [29] Lanc., Dec. 21. [30] Lanc., Jan. 18. [31] B. M. J. [32] B. M. J., Feb. 18. [33] St. Barts. Hos. J., Jan. [34] Annals Sur., Jan. [35] N. Y. M. J., Jan. 18. [36] Lanc, March 7. [37] Med. Week, Jan. 31. [38] Med. Press, March 4.

